# Tibial genu varum and primary cam morphology in healthy young adults: A cross-sectional study uncovering the double threat to joint health

**DOI:** 10.1016/j.ocarto.2026.100803

**Published:** 2026-04-28

**Authors:** Eva A. Bax, William Colyn, Johan Bellemans, Harrie Weinans, Rintje Agricola, Fleur Boel

**Affiliations:** a Department of Orthopaedic Surgery, University Medical Center Utrecht, the Netherlands; b Faculty of Medicine and Life Sciences, Hasselt University, Diepenbeek, Belgium; c GRIT Belgian Sports Clinic, Leuven, Belgium; d Department of Orthopedic Surgery, AZ Turnhout, Turnhout, Belgium; e Department of Biomechanical Engineering, Faculty 3mE, TU Delft, the Netherlands; f Department of Orthopaedics and Sports Medicine, Erasmus Medical Center, Rotterdam, Zuid-Holland, the Netherlands

**Keywords:** Hip morphology, Lower limb malalignment, Osteoarthritis, Sports participation

## Abstract

**Objectives:**

This study investigated the association between alpha angles of the hip and tibial genu varum in a healthy population with equal male-to-female distribution. It also examined sex-based differences, explored the impact of sports participation, and assessed the interplay between these conditions.

**Methods:**

Tibial, femoral, intra-articular knee deformities, and the alpha angle of the hip were analysed in 200 healthy volunteers (400 legs) aged 20–27 years using weight-bearing radiographs. The Tegner score was retrospectively collected and used to distinguish between high and low sports activity. Generalized estimating equations were used to examine the association between lower limb malalignment and alpha angle, accounting for side and gender.

**Results:**

Tibial alignment was associated with the alpha angle (β = −0.02, P = 0.002); tibial genu varum was associated with a higher alpha angle. Other deformities and their interaction with sports activity had no association with the alpha angle. Males exhibited a higher alpha angle (β = 0.19, P < 0.001, Δ = 9.0°) and more tibial genu varum (β = −0.95, P = 0.002, Δ = 1.1°) than females. High sports activity was associated with increased tibial genu varum (β = −0.75, P = 0.02) compared to low sports activity.

**Conclusion:**

This study found a significant association between alpha angle and tibial genu varum. Males exhibited higher alpha angles and more tibial genu varum than females. While higher sports activity was associated with tibial genu varum, no differences in alpha angle were seen across activity levels. These findings urge for future research to further explore mechanical load adjustments that prevent genu varum and primary cam morphology, reducing osteoarthritis risk.

## 1 Introduction

Cam morphology, characterized by an aspherical shape of the femoral head due to abnormal bone formation at the anterolateral head-neck junction [1], significantly increases the risk of developing hip osteoarthritis [[2], [3], [4]]. Cam morphology mostly develops gradually during adolescence, particularly in young male athletes engaged in high impact sports and is then referred to as primary cam morphology [5,6]. This morphology is believed to emerge in response to repetitive mechanical loading, which redistributes stress and stimulates extra bone growth [7,8], and stabilizes after closure of the proximal femoral growth plate [7].

The same phenomenon is recognized in the tibia, where genu varum is more commonly observed in high impact athletes than in non-athletes [[9], [10], [11], [12]]. Genu varum increases medial knee load and is a predictive factor for the development of knee osteoarthritis [[13], [14], [15]]. High sports participation during youth have been linked to the development of genu varum in the proximal tibia by the end of growth [10].

Cam morphology and genu varum appear to develop in response to mechanical loading during late skeletal growth, just before physeal closure [7,10]. Despite this expected shared developmental mechanism, the interplay between cam morphology and genu varum has not yet been investigated. Therefore, the primary objective of this study was to investigate the association between alpha angles of the hip and genu varum in a healthy population with equal male-to-female distribution. We hypothesize that alpha angle is associated with tibial genu varum, given their shared developmental mechanisms. The secondary objectives were to examine sex-based differences in alpha angle and genu varum, and to explore the potential influence of sports participation on these variations. Clinically, both cam morphology and genu varum are associated with an increased risk of developing hip and knee osteoarthritis, respectively. Demonstrating a significant association between these conditions would provide important insights into possible shared developmental mechanisms and suggest that modifying mechanical load during skeletal growth could help prevent these structural changes [7,10]. This may ultimately contribute to the prevention of hip and knee joint pathologies, potentially through overlapping preventive strategies, which is increasingly important given the rising incidence and prevalence of osteoarthritis [16].

## 2 Methods

### 2.1 Participants

A total of 200 healthy young adults, aged between 20 and 27 years, participated in this cross-sectional study [10]. The study was conducted from October 2009 to March 2010. Participants were recruited as volunteers from movie theatres, technical high school and university campuses, and job recruitment bureaus. Eligibility criteria required no prior history of orthopaedic issues or trauma. The study group consisted of 100 males and 100 females. Participants were retrospectively surveyed regarding their sports activities during their growth period. All participants provided written informed consent, and the study protocol was reviewed and approved by the institutional ethics committee of the University of Leuven, Belgium (B32220097076) prior to commencement.

### 2.2 Radiographic imaging protocol

All participants underwent weight-bearing full-leg radiography following the protocol outlined by Paley [17]. Radiographs were obtained with participants standing barefoot, feet together in the “stand at attention” position, and patellae facing forward. The X-ray beam was aligned with the knee, and the radiography tube was positioned 305 cm away. Radiographic parameters were set to 500 mA and 75 kV, with individual adjustments made when necessary.

### 2.3 Radiographic measurements

**Fig. 1:**
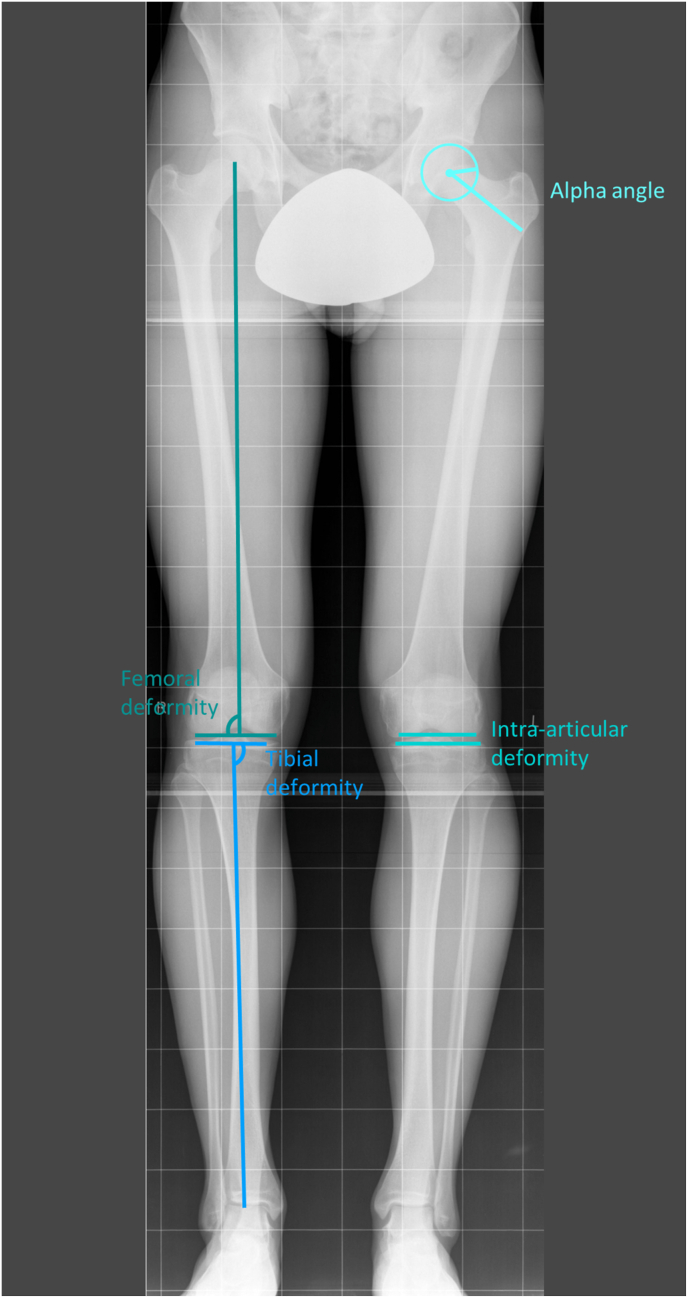
**-** Measurements of tibial, femoral, and intra-articular knee deformities, along with the alpha angle of the knee, on weight-bearing full-leg radiographs.

Radiological lower limb malalignment consists of three components: the mechanical medial proximal tibial angle (mMPTA), the mechanical lateral distal femoral angle (mLDFA), and the joint line convergence angle (JLCA) (Fig. 1). The mMPTA quantifies tibial plateau alignment and was defined as the angle between the mechanical axis of the tibia and the proximal tibial joint line [17] (Fig. 1). A neutral mMPTA ranges from 85° to 90°, with tibial genu varum alignment < 85° and tibial genu varum alignment as > 90°. The mLDFA measures femoral alignment and is defined as the angle between the mechanical axis of the femur and the distal femoral joint line [17] (Fig. 1). A neutral mLDFA ranges from 85° to 90°, with femoral genu varum alignment > 90° and femoral genu valgum alignment as < 85°.The JLCA evaluated knee joint congruity and was defined as the angle between the femoral and tibial joint lines [17] (Fig. 1). Normal JLCA values range between 0° and 2°, with values > 2° indicating an intra-articular genu varum alignment and values < 0° indicating an intra-articular genu valgum alignment. All radiographs were calibrated, and a blinded examiner conducted all measurements of lower limb alignment using the AGFA PACS software (Agfa-Gevaert).

The alpha angle, quantifies femoral head sphericity (Fig. 1). It is defined by the intersection of the femoral head-neck axis and a line from the femoral head center to the alpha point—the first deviation of the femoral head-neck junction from the best-fitting circle. The alpha angle was automatically determined on the weight-bearing full-leg radiograph using a validated method [18]. Primary cam morphology was quantified using an alpha angle ≥60° [19].

### 2.4 Athletic activity during adolescence

Participants were retrospectively surveyed about their sports activities during childhood and adolescence, categorized into three age groups. These age groups account for sex-specific growth stages: 10–12, 13–14, and 15–17 years for males, and 8–10, 11–12, and 13–15 years for females. Sports activities were quantified using the Tegner activity scale [20], a validated instrument ranging from 0 (disability due to knee problems) to 10 (participation in competitive elite-level sports). Scores of 7 or higher were classified as high-activity athletes, whereas scores below 7 indicated low-activity athletes.

Participants were then grouped based on time-varying covariates [21], which is a method that accounts for changes in activity levels over time. They were categorized into four exposure patterns: high sport activity throughout childhood, low sport activity throughout childhood, high sport activity transitioning to low sport activity, and low sport activity transitioning to high sport activity. Additionally, participants were asked whether they played soccer. For this specific group of soccer players, radiographic measurements—including the mMPTA, mLDFA, JLCA, and alpha angle —were compared to those of non-soccer players and non-soccer players with high levels of sports activity.

### 2.5 Statistical analysis

All statistical analyses were performed using SPSS version 27.0 (IBM, Armonk, NY) in accordance with the Checklist for Statistical Assessment of Medical Papers [22]. Variables were assed for normality using histograms. Descriptive statistics, including means and standard deviations for normally distributed data, and medians with interquartile ranges for non-normally distributed data, were provided. A generalized estimating equation model with a gamma distribution was used to analyse the alpha angle (dependent variable), considering mMPTA, mLDFA, JLCA, gender, and sports activity as independent variables. Side was included in the model to adjust for correlations in within subject variables, using an unstructured working correlation matrix. Additionally, the effect of gender and sport activity on alpha angle and mMPTA was examined, with side included to account for correlations between repeated measurements. Statistical significance was defined as a p-value <0.05.

## 3 Results

### 3.1 Patient characteristics

The study included 100 males and 100 females. The mean BMI was 22.1 ± 3.1 kg/m^2^. The median alpha angle was 46.1° (IQR 10.4°). Of the participants, 54 legs (13.5%) had an alpha angle ≥60° (median alpha angle 67.2°, interquartile ranges 12.8°). The mean mMPTA was 84.1 ± 2.1°, mLDFA was 88.0 ± 1.8°, and JLCA was 0.3 ± 1.1°. Of the participants, 66 had tibial genu varum (mean mMPTA 83.8 ± 1.0°), and 26 legs had tibial genu valgum (mean mMPTA 91.0 ± 0.9°). Femoral genu valgum was present in 19 legs (mean mLDFA 84.1 ± 0.7°), while femoral genu varum was found in 42 legs (mean mLDFA 91.0 ± 1.6°). Additionally, 21 legs showed intra-articular genu varum (mean JLCA 2.5 ± 0.5°), and 117 legs had intra-articular genu valgum (mean JLCA -1.1 ± 0.8°).

### 3.2 Sport activity

Regarding sport activity levels, the mean Tegner score during the second decade of life was 6.3 ± 2.3 for males and 4.8 ± 1.8 for females. Of the participants, 92 participants (46.0%) had low sport activity, 68 (34.2%) had high sport activity, 23 (11.6%) switched from high to low activity, and 17 (8.5%) increased their activity levels during childhood. Among male participants, 54 (54.0%) did not play soccer during childhood, with 12 males (12.0%) having high activity based on the Tegner score. 31 males (31.0%) played soccer throughout this period, all with high activity, except for one. Ten males (10.0%) discontinued playing soccer during childhood, while five (5.0%) started. Soccer players showed a lower mMPTA, when compared to non-soccer players and male non-soccer players with a high level of sports activity during childhood (Table 1). Furthermore, soccer players exhibit a higher alpha angle of the hip than both non-soccer players and male non-soccer players with a high sports activity level during childhood (Table 1). Female soccer players were excluded from these analyses due to the limited sample size, as only two women had played soccer during childhood.

**Table 1:** Radiological measurements for male soccer players (N = 31 (31%), 62 legs), male non-soccer players (N = 54 (54%), 108 legs), and male non-soccer players with a high sports activity level (N = 12 (12%), 24 legs). mMPTA, mLDFA, and JLCA are presented as mean ± SD. Alpha angle is presented as median (IQR).

	mMPTA	mLDFA	JLCA	Alpha Angle
Male soccer players	85.4 °± 2.2 °	87.9 °± 1.8 °	0.14 °± 1.2 °	52.4 ° (16.0 °)
Male non-soccer players	87.0 °± 1.7 °	87.9 °± 2.2 °	0.38 °± 1.1 °	50.7 ° (9.3 °)
Male non-soccer players high sport activity level	86.7 °± 2.7 °	88.3 °± 1.2 °	0.3 °± 1.2 °	49.1 ° (6.2 °)

mMPTA, mechanical medial proximal tibial angle; mLDFA, mechanical lateral distal femoral angle; JLCA, joint line convergence angle.

### 3.3 Association between gender, sport activity, and radiographic measurements

**Fig. 2:**
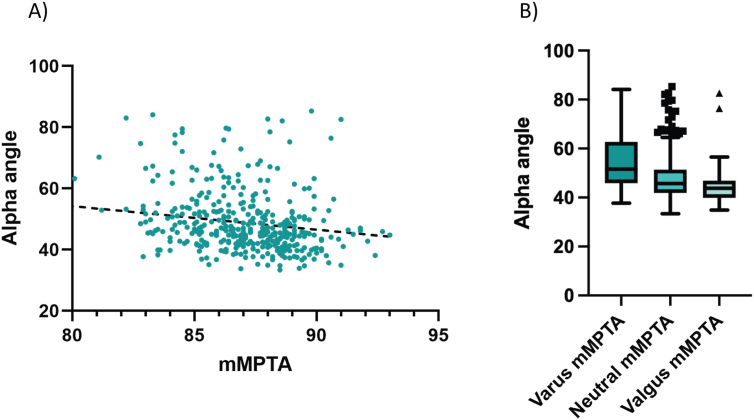
Relationship between medial proximal tibial angle (mMPTA) and the alpha angle. A) Scatter plot showing the relationship between the mMPTA and the alpha angle, demonstrating a significant association. B) Boxplot of alpha angles for the three tibia groups: genu varum (66 lower limbs), neutral (308 lower limbs), and genu valgum (26 lower limbs).

The generalized estimating equation analysis, accounting for side, revealed that the mMPTA had a significant association with alpha angle (β = −0.02, 95% CI [−0.03, −0.01], P = 0.002) (Fig. 2A). This indicates that a lower mMPTA, representing increased tibial genu varum, was associated with a higher alpha angle. The same trend was observed when comparing the three tibia groups, with tibial genu varum mMPTA showing higher alpha angles than the neutral group (Fig. 2B). In contrast, neither the mLDFA (β = −0.01, CI [−0.02, 0.00], P = 0.21) nor the JLCA (β = 0.01, CI [−0.01, 0.02], P = 0.48) showed a significant relationship with the alpha angle. Furthermore, the interaction terms between exposure patterns of sport activity and mMPTA, mLDFA, and JLCA were not statistically significant.

**Fig. 3:**
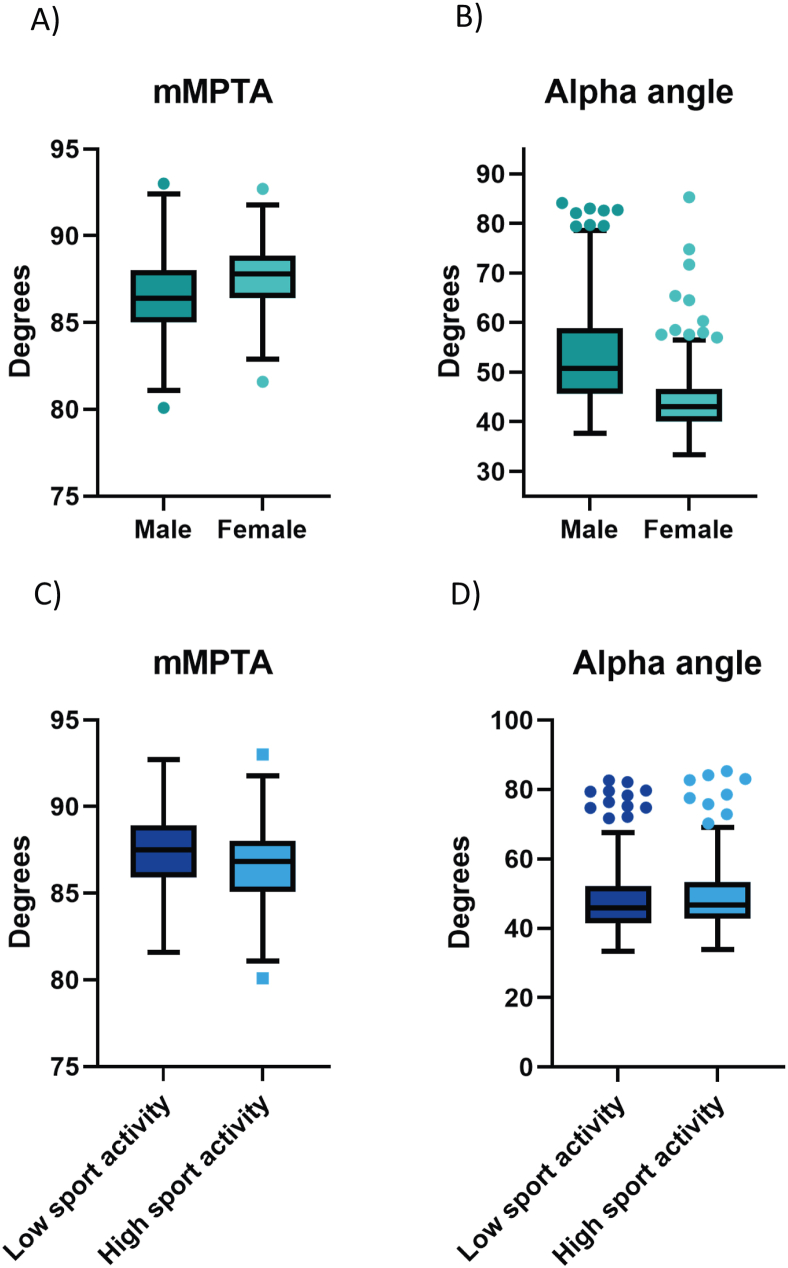
Differences in radiographic measurements between males and females, as well as between low and high sport activity groups. (A) mMPTA comparison between males and females. (B) Alpha angle comparison between males and females. (C) mMPTA comparison between low- and high-activity athletes (D) Alpha angle comparison between low- and high-activity athletes.

Males show a significantly lower mMPTA than females (β = −0.95, CI [−1.56, −0.34], P = 0.002), accounting for side and sport activity (Fig. 3A). Males also have a significantly higher alpha angle than females (β = 0.19, CI [0.14, 0.23], P < 0.001) (Fig. 3B). High sports activity was associated with a lower mMPTA compared to low sport activity (β = −0.75, CI [−1.37, −0.13], P = 0.02), accounting for gender and side (Fig. 3C). No statistically significant difference in alpha angle and sports activity was found (β = −0.01, CI [−0.05, 0.04], P = 0.79) (Fig. 3D).

## 4 Discussion

This study investigated the association between hip alpha angles and tibial genu varum in a healthy population with an equal male-to-female distribution. The most important finding of this study was the significant association between alpha angle and tibial genu varum, with a lower mMPTA linked to a higher alpha angle. No statistically significant interaction with sport activity was detected, suggesting that mMPTA’s influence on alpha angle is not affected by activity level. Both primary cam morphology and tibial genu varum are associated with an increased risk of hip and knee osteoarthritis [[2], [3], [4],^13^], respectively, indicating the potential clinical importance of their association. Additionally, males had significantly higher alpha angle values and lower mMPTA compared to females, reinforcing that primary cam morphology and tibial genu varum are more common in males [5,[23], [24], [25], [26], [27], [28]].

Current literature highlights the association between primary cam morphology and high-impact sports, particularly soccer, likely due to the repetitive mechanical stress on the hip during adolescence [2,5,7,8,29]. Our study found no significant differences in alpha angle between high and low sport activity levels during childhood. However, male soccer players had a higher alpha angle compared to male non-soccer players and male non-soccer players with high sport activity levels. The literature further supports the association between involvement in sports, and greater tibial genu varum, emphasizing the role of physical activity in shaping lower leg alignment [[9], [10], [11], [12],^15^]. Notably, lower leg alignment has been widely acknowledged as a contributing factor to the risk of sports injuries [30]. Our study also showed that participants with high sport activity during childhood had a significantly lower mMPTA compared to those with low sport activity, while accounting for gender in a balanced male and female population. Male soccer players had a lower mMPTA compared to male non-soccer players and male non-soccer players with high sport activity. Despite these findings, the direct relationship between tibial genu varum and alpha angle has been underexplored. Our research addresses this gap by demonstrating a significant association between tibial genu varum and a cam shaped femoral head.

Our study demonstrates an association, not a causal relationship, between alpha angle and tibial genu varum. While causality implies direct cause-and-effect, our findings show a statistical connection without confirming direct causality. Klij et al. [31] suggested that there is no causal relationship between cam morphology and genu varum orientation, as these features appear to develop simultaneously at the last phase of growth. It has been suggested that children with bowlegs may have a functional advantage in sports like soccer, potentially due to natural selection [10]. Mechanical studies, for example Hueter-Volkmann's law, explain how loading affects knee growth [[32], [33], [34], [35]]. Primary cam morphology typically develops gradually during skeletal maturation in response to repetitive mechanical loading, stabilizing after the closure of the proximal femoral growth plate [5,7]. In young soccer players, the primary cam morphology becomes apparent once the growth plate closes [5], emphasizing the role of sport-specific loading. In addition, previous studies suggest that lower-limb alignment and hip morphology are interconnected within the kinetic chain. Frontal knee alignment has been associated with hip morphology and hip pain [36], and alterations in frontal knee alignment may also lead to changes in frontal hip alignment [37]. Furthermore, hip shape has been linked to knee osteoarthritis, and femoral morphology has been associated with the development of knee pain [38], highlighting the complex biomechanical relationship between hip structure and knee joint pathology [39]. Our findings suggest that both cam morphology and genu varum alignment may develop through similar adaptive mechanisms of the growth plates in response to mechanical loading. Consequently, the observed association may partly reflect shared developmental factors rather than a direct biological link. Clinically, both cam morphology and genu varum have been associated with increased risks of hip and knee osteoarthritis, respectively. Understanding their relationship may provide insight into these shared mechanisms and help guide strategies to modify mechanical load during growth, potentially reducing the risk of future joint pathology [16]. Further research is needed to explore how mechanical load adjustments during growth might prevent the development of tibial genu varum and cam morphology, potentially reducing the future risk of osteoarthritis.

Primary cam morphology and tibial genu varum are associated with an increased risk of hip [[2], [3], [4]] and knee osteoarthritis [14,15,40,41], posing a significant threat to joint health. Osteoarthritis is a major global health burden with rising healthcare costs [16,42]. Aging and obesity, key risk factors [43,44], are increasing, with obesity expected to reach 50% by 2030 [45,46] and the elderly population continuing to grow [47]. As a result, osteoarthritis prevalence will rise further [16]. Targeted prevention strategies during skeletal growth could potentially reduce osteoarthritis incidence. Adjusting mechanical loads on the knees in males may help prevent tibial genu varum alignment and its associated structural changes [7,10]. Similarly, modifying athletic activities during critical growth periods could influence the development of primary cam morphology [7], which may contribute to reducing osteoarthritis prevalence. Therefore, future research should investigate preventive strategies that may reduce the incidence of both hip and knee osteoarthritis in athletes, contributing to long-term joint health and reducing healthcare burdens.

This study has several limitations. Its retrospective design may have introduced recall bias, particularly regarding self-reported sports participation during adolescence. Furthermore, the radiographic assessments of lower limb alignment were conducted by a single observer who was blinded to additional information. The accuracy of the observer may influence these measurements, potentially leading to systematic bias. However, previous studies have shown that both intra- and interobserver reliability are excellent, with reported values exceeding 0.85 for these measurements [48,49]. The alpha angle measurement accuracy depends on accurate landmark placement. The automatic search model was trained on a different dataset consisting of primarily anteroposterior pelvic radiographs. However, the training set of the automatic search model also contained long-limb radiographs like those used in the current study. Therefore, we think that the automated method could be used appropriately on the weight-bearing full-leg radiograph. Another limitation is the use of weight-bearing radiographs, where knee positioning may affect the reliability of the measurements [48,50,51]. We mitigated this by standardizing limb rotation to ensure forward facing patellae [17]. However, detection of primary cam morphology on weight-bearing full-leg radiographs is limited by projection: cam morphology is typically located anterolaterally on the femoral head-neck junction and is best visualized with the hip in slight internal rotation or on dedicated lateral views. Since weight-bearing full-leg radiographs are taken in a neutral position and no lateral hip views were available, the prevalence of primary cam morphology in this cohort is likely underestimated. In addition, the cross-sectional study design precludes any causal inference, and the study cohort consisted of healthy young adults, which may limit generalizability to symptomatic populations with femoroacetabular impingement. Finally, no power calculation was performed for the current study objectives.

In conclusion, this study investigated the association between lower limb malalignment and alpha angle in a healthy participant population with an equal male-to-female distribution. We found a significant association between alpha angle and tibial genu varum, with genu varum linked to a higher alpha angle. Males exhibited higher alpha angles and more tibial genu varum than females. While higher sports activity was associated with a tibial genu varum, alpha angle and femoral or intra-articular knee deformities showed no difference across activity levels. Future research should focus on mechanical load adjustments in at-risk individuals to potentially prevent leg malalignment and cam morphology, which may help reduce the incidence of knee and hip osteoarthritis and improving long-term joint health.

## Informed consent

Informed consent was obtained from all individual participants included in the study.

## Ethical approval

The study was approved by an Ethics Committee of the University of Leuven, Belgium (B32220097076).

## Contributions

Study conception and design: EB, HW, RA, FB.

Acquisition of data: WC, JB.

Analysis & interpretation of data: EB, FB.

Writing of first manuscript draft: EB.

Critical manuscript revision and approval of final manuscript: All authors.

EB, RA, and FB had full access to all of the data in the study and takes responsibility for the integrity of the data and the accuracy of the data analysis.

## Conflicts of interest

EB, JB, RA, and FB declare no conflicts of interest.

WC reports receiving payment or honoraria for lectures, presentations, speakers bureaus, manuscript writing, or educational events from Smith & Nephew.

HW reports research grants or contracts from Interreg (EFRO), IMI-APPROACH, OA-Inject (NWO), 3DHip (Eurostars), Dartbac (NWO), LUMINATE (EU), SHIELD (Eurostars), and Kansen voor West (Province of Utrecht), all through UMC Utrecht and not directly related to the current work. HW is also a minority shareholder of Replasia, Presurgeo, Amation, and Preimure.

## Abbreviations

IQR: Interquartile ranges
JLCA: Joint line convergence angle
mLDFA: Mechanical lateral distal femoral angle
mMPTA: Mechanical medial proximal tibial angle
SD: Standard deviation

## References

[1] Ito K., Minka-Ii M.A., Leunig M., Werlen S., Ganz R. (2001). Femoroacetabular impingement and the cam-effect. J. Bone Joint Surg..

[2] Agricola R., Waarsing J.H., Arden N.K. (2013). Cam impingement of the hip—a risk factor for hip osteoarthritis. Nat. Rev. Rheumatol..

[3] Tang J., van Buuren M.M.A., Riedstra N.S. (2023). Cam morphology is strongly and consistently associated with development of radiographic hip osteoarthritis throughout 4 follow-up visits within 10 years. Osteoarthr. Cartil..

[4] Casartelli N.C., Maffiuletti N.A., Valenzuela P.L. (2021). Is hip morphology a risk factor for developing hip osteoarthritis? A systematic review with meta-analysis. Osteoarthr. Cartil..

[5] Agricola R., Bessems J.H.J.M., Ginai A.Z. (2012). The development of cam-type deformity in adolescent and young Male soccer players. Am. J. Sports Med..

[6] Dijkstra H.P., Mc Auliffe S., Ardern C.L. (2023). Oxford consensus on primary cam morphology and femoroacetabular impingement syndrome: part 1—definitions, terminology, taxonomy and imaging outcomes. Br. J. Sports Med..

[7] Agricola R., Heijboer M.P., Ginai A.Z. (2014). A cam deformity is gradually acquired during skeletal maturation in adolescent and young Male soccer players. Am. J. Sports Med..

[8] Roels P., Agricola R., Oei E.H., Weinans H., Campoli G., Zadpoor A.A. (2014). Mechanical factors explain development of cam-type deformity. Osteoarthr. Cartil..

[9] Thijs Y., Bellemans J., Rombaut L., Witvrouw E. (2012). Is high-impact sports participation associated with bowlegs in adolescent boys?. Med. Sci. Sports Exerc..

[10] Colyn W., Agricola R., Arnout N., Verhaar J.A.N., Bellemans J. (2016). How does lower leg alignment differ between soccer players, other athletes, and non-athletic controls?. Knee Surg. Sports Traumatol. Arthrosc..

[11] Witvrouw E., Danneels L., Thijs Y., Cambier D., Bellemans J. (2009). Does soccer participation lead to genu varum?. Knee Surg. Sports Traumatol. Arthrosc..

[12] Asadi K., Mirbolook A., Heidarzadeh A., Kivi M.M., Meybodi M.K.E., Rad M.R. (2015). Association of soccer and genu varum in adolescents. Trauma Mon..

[13] Tanamas S., Hanna F.S., Cicuttini F.M., Wluka A.E., Berry P., Urquhart D.M. (2009). Does knee malalignment increase the risk of development and progression of knee osteoarthritis? A systematic review. Arthritis Care Res (Hoboken).

[14] Sharma L., Chmiel J.S., Almagor O. (2013). The role of varus and valgus alignment in the initial development of knee cartilage damage by MRI: the MOST study. Ann. Rheum. Dis..

[15] Brouwer G.M., Tol AW Van, Bergink A.P. (2007). Association between valgus and varus alignment and the development and progression of radiographic osteoarthritis of the knee. Arthritis Rheum..

[16] Cui A., Li H., Wang D., Zhong J., Chen Y., Lu H. (2020). Global, regional prevalence, incidence and risk factors of knee osteoarthritis in population-based studies. EClinicalMedicine.

[17] Paley D. (2002). Principles of Deformity Correction.

[18] Boel F., de Vos-Jakobs S., Riedstra N.S. (2024). Automated radiographic hip morphology measurements: an open-access method. Osteoarthritis Imaging.

[19] Mascarenhas V.V., Castro M.O., Afonso P.D. (2021). The Lisbon agreement on femoroacetabular impingement imaging—part 2: general issues, parameters, and reporting. Eur. Radiol..

[20] Tegner Y., Lysholm J. (1985). Rating systems in the evaluation of knee ligament injuries. Clin. Orthop. Relat. Res..

[21] Meinert R., Frischer T., Kuehr J. (1994). Assessing the effect of time-varying covariates in cross-sectional studies. J. Clin. Epidemiol..

[22] Mansournia M.A., Collins G.S., Nielsen R.O. (2021). CHecklist for statistical assessment of medical papers: the CHAMP statement. Br. J. Sports Med..

[23] Bellemans J., Colyn W., Vandenneucker H., Victor J. (2012).

[24] Tang J., van Buuren M.M.A., Boel F. (2024). The association between cam morphology and hip pain in males and females within 10 years: a national prospective cohort study (CHECK). Semin. Arthritis Rheum..

[25] Hwang D., Wook Choi M., Kim S.H. (2023). Age and sex differences in coronal lower extremity alignment in a healthy Asian population. Knee.

[26] Siboni R., Vialla T., Joseph E. (2022). Coronal and sagittal alignment of the lower limb in Caucasians: analysis of a 3D CT database. Orthop. Traumatol.: Surgery and Research.

[27] Alter T.D., Knapik D.M., Lambers F. (2022). Sex-based differences in femoroacetabular impingement syndrome and the effect of cam deformity location on the extent of labral tearing: a 3-Dimensional computed tomography study. Orthop. J. Sports Med..

[28] Ahedi H., Winzenberg T., Bierma-Zeinstra S. (2022). A prospective cohort study on cam morphology and its role in progression of osteoarthritis. Int J Rheum Dis.

[29] Pettit M., Doran C., Singh Y., Saito M., Sunil Kumar K.H., Khanduja V. (2021). How does the cam morphology develop in athletes? A systematic review and meta-analysis. Osteoarthr. Cartil..

[30] Jan van de Pol G., Arnold M.P., Verdonschot N., van Kampen A. (2009). Varus alignment leads to increased forces in the anterior cruciate ligament. Am. J. Sports Med..

[31] van Klij P., Heijboer M.P., Ginai A.Z., Verhaar J.A.N., Waarsing J.H., Agricola R. (2021). Clinical and radiological hip parameters do not precede, but develop simultaneously with cam morphology: a 5-year follow-up study. Knee Surg. Sports Traumatol. Arthrosc..

[32] Volkmann R. Chirurgische Erfahrungen uber Knochenverbiegung und Knochenwachtsum. Virchows Arch Pathol Anat. Published online 1862:512-540.

[33] Willy C., Schneider P., Engelhardt M., Hargens A.R., Mubarak S.J. (2008). Richard von Volkmann. Clin. Orthop. Relat. Res..

[34] Hueter C. XXIII. Anatomische Studien an Den Extremitiitengelenke. Neugeborener Und Erwachsener.

[35] Eastwood D.M., Sanghrajka A.P. (2011). Guided growth. J Bone Joint Surg Br.

[36] Ahmad S.S., Kerber V., Konrads C. (2021). The ischiofemoral space of the hip is influenced by the frontal knee alignment. Knee Surg. Sports Traumatol. Arthrosc..

[37] Patel J., Patel R., Melton J. (2023). Changes in coronal alignment of the hip joint after medial opening wedge high tibial osteotomy. Eur. J. Orthop. Surg. Traumatol..

[38] Nelson A.E., Golightly Y.M., Renner J.B. (2016). Variations in hip shape are associated with radiographic knee osteoarthritis: cross-sectional and longitudinal analyses of the Johnston county osteoarthritis project. J. Rheumatol..

[39] Kaizu Y., Miyata K., Arii H., Tazawa M., Yamaji T. (2021). Femoral morphology is associated with development of knee pain after hip fracture injury among older adults: a nine-year retrospective study. J. Orthop..

[40] Sharma L., Song J., Dunlop D. (2011). Varus and valgus alignment and incident and progressive knee osteoarthritis. Annuals of the Rheumatic Diseases.

[41] Miyazaki T., Wada M., Kawahara H., Sato M., Baba H., Shimada S. (2002). Dynamic load at baseline can predict radiographic disease progression in medial compartment knee osteoarthritis. Ann. Rheum. Dis..

[42] Hunter D., Bierma-Zeinstra S. (2019). Osteoarthritis. Lancet.

[43] Georgiev T., Angelov A.K. (2019). Modifiable risk factors in knee osteoarthritis: treatment implications. Rheumatol. Int..

[44] Driban J.B., Harkey M.S., Barbe M.F. (2020). Risk factors and the natural history of accelerated knee osteoarthritis: a narrative review. BMC Muscoskelet. Disord..

[45] Malik V.S., Willet W.C., Hu F.B. (2020). Nearly a decade on — trends, risk factors and policy implications in global obesity. Nat. Rev. Endocrinol..

[46] Mehrzad R. (2020). Obesity: Global Impact and Epidemiology.

[47] Grinin L., Grinin A., Korotayev A. (2023). Global aging and our futures. World Futures.

[48] Nguyen H.C., Gielis W.P., van Egmond N. (2021). The need for a standardized whole leg radiograph guideline: the effects of knee flexion, leg rotation, and X-ray beam height. Journal of Cartilage & Joint Preservation.

[49] Nguyen H.C., van Egmond N., Hevesi M., Weinans H., Gielis W.P., Custers R.J.H. (2022). A new protocol for obtaining whole leg radiographs with excellent reproducibility. Journal of Cartilage and Joint Preservation.

[50] Cooke T.D.V., Sled E.A., Scudamore R.A. (2007). Frontal plane knee alignment: a call for standardized measurement. J. Rheumatol..

[51] Specogna A.V., Birmingham T.B., Hunt M.A. (2007). Radiographic measures of knee alignment in patients with varus gonarthrosis. Am. J. Sports Med..

